# Advances in Understanding the Role of NRF2 in Liver Pathophysiology and Its Relationship with Hepatic-Specific Cyclooxygenase-2 Expression

**DOI:** 10.3390/antiox12081491

**Published:** 2023-07-26

**Authors:** Marina Fuertes-Agudo, María Luque-Tévar, Carme Cucarella, Paloma Martín-Sanz, Marta Casado

**Affiliations:** 1 Instituto de Biomedicina de Valencia (IBV), CSIC, Jaume Roig 11, 46010 Valencia, Spain; mfuertes@ibv.csic.es (M.F.-A.); mluque@ibv.csic.es (M.L.-T.); cucarella@ibv.csic.es (C.C.); 2 Centro de Investigación Biomédica en Red de Enfermedades Hepáticas y Digestivas (CIBERehd), Monforte de Lemos 3-5, 28029 Madrid, Spain; 3 Instituto de Investigaciones Biomédicas (IIB) “Alberto Sols”, CSIC-UAM, Arturo Duperier 4, 28029 Madrid, Spain

**Keywords:** liver diseases, NRF2, COX-2, oxidative stress, inflammation

## Abstract

Oxidative stress and inflammation play an important role in the pathophysiological changes of liver diseases. Nuclear factor erythroid 2-related factor 2 (NRF2) is a transcription factor that positively regulates the basal and inducible expression of a large battery of cytoprotective genes, thus playing a key role in protecting against oxidative damage. Cyclooxygenase-2 (COX-2) is a key enzyme in prostaglandin biosynthesis. Its expression has always been associated with the induction of inflammation, but we have shown that, in addition to possessing other benefits, the constitutive expression of COX-2 in hepatocytes is beneficial in reducing inflammation and oxidative stress in multiple liver diseases. In this review, we summarized the role of NRF2 as a main agent in the resolution of oxidative stress, the crucial role of NRF2 signaling pathways during the development of chronic liver diseases, and, finally we related its action to that of COX-2, where it appears to operate as its partner in providing a hepatoprotective effect.

## 1. Introduction

In 1956, Harman described the free radical theory [1]. Since then, reactive oxygen species (ROS) have been proposed as the main cause of aging and inflammatory diseases. ROS include both free radicals and oxygen intermediates, such as superoxide radical (O_2^•−^_), hydrogen peroxide (H_2_O_2_), hydroxyl radicals (**^•^**OH) and singlet oxygen (^1^O_2_). The main source of ROS in vivo is aerobic respiration [2]. However, ROS are also produced by peroxisomal β-oxidation of fatty acids [3], in the metabolism of xenobiotics by cytochrome P450 [4], during the stimulation of phagocytosis by pathogens or lipopolysaccharide (LPS) through nicotinamide adenine dinucleotide phosphate (NADPH) oxidases [5], and/or by the activity of tissue-specific cellular enzymes such as cyclooxygenase [6].

ROS levels are tightly regulated and normally contribute to cellular and tissue homeostasis as signaling molecules. Conversely, an excess of ROS generation and/or impaired antioxidant activity under acute and chronic oxidative stress are associated with metabolic “reprogramming,” inflammation, tissue damage/dysfunction, and toxicity, leading to senescence or death [7]. Thus, the free radical theory was replaced by the redox hypothesis in which oxidative stress is defined as the imbalance between oxidant and antioxidant mechanisms in favor of the former, triggering a disturbance in oxidation-reduction reactions which leads to cellular damage through the oxidation of DNA, RNA, carbohydrates, proteins, and lipids [8].

When considering the dual role of ROS from their physiological role as second messengers, to their involvement in inflammation and tissue damage, it is necessary for cells to have mechanisms that regulate redox homeostasis, maintaining antioxidant, anti-inflammatory and detoxification responses. Nuclear factor erythroid 2-related factor 2 (NRF2) regulates the expression of multiple antioxidant and cytoprotective proteins and enzymes and is considered the main mediator of cellular adaptation to redox stress [9].

Cyclooxygenases (COX-1, COX-2) are key actors in the biosynthesis of prostanoids. *PTGS1*, the COX-1-encoding gene, is constitutively expressed in many tissues, whereas *PTGS2* (prostaglandin synthase), the COX-2-encoding gene, is expressed and induced by different stimuli in several tissues and cellular types; however, in the liver, COX-2 expression is restricted to those situations in which proliferation or de-differentiation occurs [10]. Thus, adult hepatocytes only express COX-2 under pathological conditions. Notwithstanding, our group has demonstrated that the constitutive COX-2 expression, specifically in hepatocytes, protects from liver injury in several models [11,12,13,14], which has allowed us to hypothesize that the induction of COX-2 plays a protective role as a physiologic response against liver injury, in part by reducing hepatic recruitment and the infiltration of neutrophils, producing a significant attenuation of oxidative stress and hepatic apoptosis, increasing autophagic flux and decreasing endoplasmic reticulum (ER) stress.

Numerous excellent reviews have been written on how NRF2 controls the cellular redox state (see for example [15]). In this review, we explore key aspects in the control of NRF2 expression and its function as a regulator of the antioxidant response, as well as other aspects beyond this function, focusing on its relevance in liver diseases. In addition, we explore the possible role it may play in the observed hepatoprotective COX-2-dependent response.

## 2. NRF2 as a Sensor of Cellular Redox State

NRF2 is continuously produced by the *NFE2L2* gene and is immediately degraded through the ubiquitin-proteasome system. This apparently futile mechanism is extremely useful in that it allows cells to respond rapidly to potentially harmful oxidative and electrophilic challenges.

**Figure 1:**
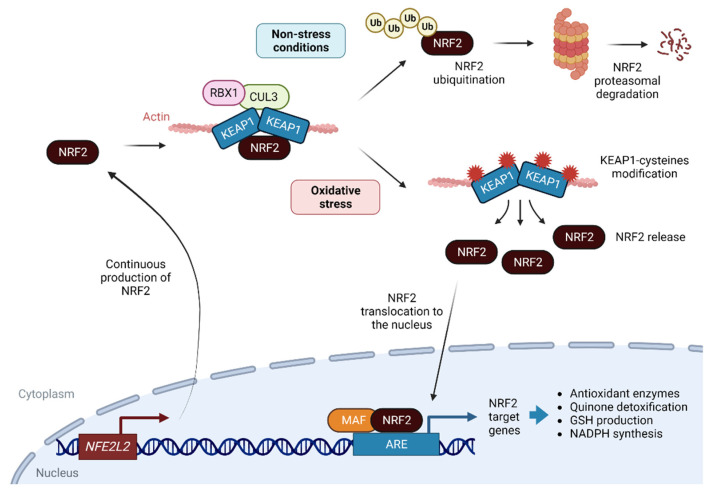
Molecular mechanisms of the KEAP1-NRF2 ARE pathway: NRF2 is continuously transcribed and translated. Under non-stress conditions, NRF2 is retained by KEAP1, together with CUL3 and RBX, ubiquitinated and degraded by the proteasome. In the context of oxidative stress, KEAP1 cysteines are modified by ROS, releasing NRF2, which translocates to the nucleus, binds to MAF proteins and induces the expression of genes involved in the antioxidant response. Abbreviations: ARE, antioxidant response elements; CUL3, cullin 3; KEAP1, Kelch-like ECH-associated protein 1; NRF2, nuclear factor-erythroid 2; *NFE2L2*, NRF2 gene; RBX1, RING-box protein 1; MAF, small musculoaponeurotic fibrosarcoma oncogene homologue; Ub, ubiquitin. Created with BioRender.com (accessed on 18 July 2023).

A canonical pathway for NRF2 stabilization and degradation is widely studied and accepted. Under non-stressed conditions, NRF2 localizes to the cytoplasm where it interacts with the actin-binding protein Kelch-like ECH associating protein 1 (KEAP1). KEAP1 is a homodimeric protein that binds NRF2 with the E3 ligase complex formed by Cullin 3 and RING-box protein 1 (CUL3/RBX1). Under homeostatic conditions, the N-terminal domain of the KEAP1 homodimer binds a molecule of NRF2 that is rapidly degraded by the ubiquitin-proteasome 26S pathway by CUL3/RBX1 [9,16]. Under oxidative stress conditions, KEAP1 is a redox and electrophilic sensor that undergoes cysteine modification, mainly C155, C273 and C288, which is critical for its ability to repress NRF2, which, after phosphorylation at serine (Ser) 40 by protein kinase Cδ (PKCγ), is translocated to the nucleus [17] (Figure 1). In the nucleus, NRF2 is a basic region leucine zipper (bZip) transcription factor that forms heterodimers with the small musculoaponeurotic fibrosarcoma (MAF) proteins K, G, and F, and recognizes an enhancer sequence called the antioxidant response element (ARE) [18]. AREs are present in the regulatory regions of more than 250 genes (ARE genes) involved in a wide range of homeostatic mechanisms related to metabolism and redox signaling, inflammation, and proteostasis [9].

From the point of view of antioxidant function, NRF2 regulates: (i) important enzymes for the production of nicotinamide adenine dinucleotide phosphate (NADPH) redox cofactor, including glucose 6-phosphate dehydrogenase (G6PD, key enzyme for carbohydrate metabolism); (ii) de novo glutathione (reduced glutathione, GSH) synthetic enzymes, including glutathione S-transferase (GST), glutathione reductase (GR), catalytic (GCLc) and modulator (GCLm) subunits of glutamate-cysteine ligase, γ-glutamylcysteine synthetase (γ-GCS) and glutathione peroxidase (GPx); (iii) key enzymes for the production of antioxidant bilirubin, including heme-oxygenase-1 (HO-1), and biliverdin reductase (BVR); (iv) direct antioxidant enzymes as thioredoxin (TRX) and superoxide dismutase (SOD); and (v) NAD(P)H:quinone oxidoreductase 1 (NQO1), an essential enzyme for quinone detoxification [19]. Moreover, NRF2 can regulate its own expression through an ARE-like element located in the proximal region of *NFE2L2* gene promoter [20]. Transcription arrest of NRF2 is also triggered by the bZIP protein BTB domain and CNC homolog 1 (BACH1), that represses the ARE sequence by heterodimerizing with MAFs proteins.

The repression of BACH1 is dominant over NRF2 activation and, for *HMOX1* transcription (HO-1-encoding gene), inactivation of BACH1 is a prerequisite for *NFE2L2* induction by allowing binding of NRF2 already present in the nucleus [21]. Many of the NRF2 target genes were identified thanks to the use of mice deficient in NFR2 (NRF2 KO) or KEAP1 (KEAP1 KO), or through the administration of small molecules that pharmacologically activate NRF2, usually through kinases that phosphorylate the transcription factor interfering with its binding to KEAP1 [22].

In addition to KEAP1 interaction, NRF2 binds to different proteins that regulate its stabilization or degradation. Thus, the coactivator CREB-binding protein (CBP) acetylates NRF2, stabilizing it, promoting its nuclear localization, and enhancing its activity [23]. In the opposite way, histone deacetylases (HDACs) type 1, and sirtuin 1 and 2 (SIRT1 and SIRT2) decrease NRF2 transcriptional activity [24].

**Figure 2:**
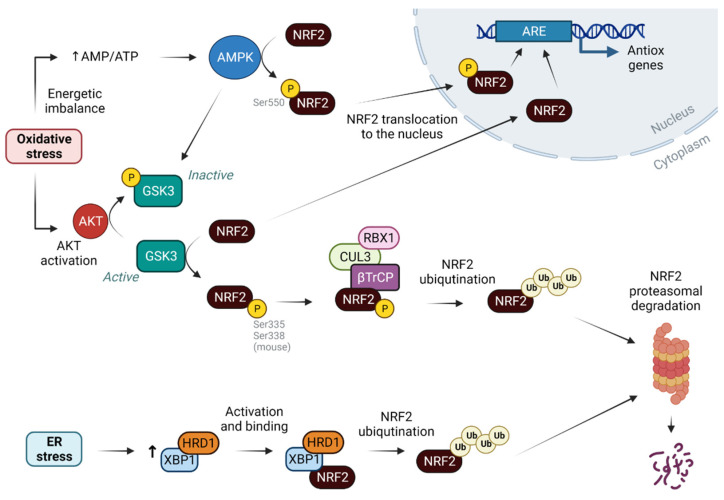
NRF2 regulation pathway independent of KEAP1: stabilization and degradation of NRF2 is mediated by different components in addition to KEAP1. Phosphorylation of NRF2 at Ser550 by AMPK leads to its translocation and stabilization in the nucleus. AMPK is activated when an imbalance in the AMP/ATP ratios occurs in the context of oxidative stress. Oxidative stress also activates AKT, thus phosphorylating GSK3, inactivating it and blocking NRF2 degradation. When GSK3 is active, it phosphorylates NRF2 at Ser335 and Ser338, targeting ubiquitination through βTrCP binding, along with CUL3 and RBX. Finally, under ER stress conditions, the XBP1/HRD1 complex can also bind NRF2 and mark it for degradation. Abbreviations: AMPK, 5′ AMP-activated protein kinase; AKT, protein kinase B; GSK3, glycogen synthase kinase 3; βTrCP, F-box/WD repeat-containing protein 1A; ER, endoplasmic reticulum; HRD1, E3 ubiquitin ligase synoviolin; XBP1, X-box binding protein 1; P, phosphate. Created with BioRender.com (accessed on 6 July 2023).

Regarding NRF2 degradation, an alternative pathway to KEAP1 is through the glycogen synthase kinase 3 (GSK-3). This kinase phosphorylates NRF2, targeting for ubiquitination upon binding to another E3 ubiquitin ligase that is the F-box/WD repeat-containing protein 1A (β-TrCP), together with the cullin (CUL) 3/ring-box protein (RBX) complex (Figure 2). Upon activation of the protein kinase B (AKT) pathway, under stress conditions for example, AKT is able to phosphorylate and inhibit GSK-3, thus inhibiting NRF2 degradation. Insulin and WNT signaling can also trigger the activation of the AKT pathway through the protein tyrosine phosphatase 1B (PTP1B) and the insulin-like growth factor 1 (IGF-1) receptor. Both pathways lead to the phosphorylation of GSK-3, prompting NRF2 activation, facts that have been demonstrated both in a model of acetaminophen hepatotoxicity [25] and in a model of cholangiocyte expansion [26]. Prior to oxidative stress, an increase in the adenosine monophosphate/adenosine triphosphate (AMP/ATP) ratio occurs, which could be sensed by the 5′ AMP-activated protein kinase (AMPK) [27]. In this situation, AMPK becomes activated and phosphorylates NRF2 at Ser550, causing its nuclear accumulation (Figure 2). In addition, AMPK inhibits GSK-3β, thus blocking NRF2 degradation [28]. Furthermore, WNT signaling controls the zonal expression of NRF2 in hepatocytes maintaining a perivenous phenotype [29].

Another degradation mechanism for NRF2 was proposed involving inositol requiring enzyme 1 (IRE1)/E3 ubiquitin ligase synoviolin (HRD1) present in the ER and whose expression is enhanced by activation of the X-box binding protein 1 (XBP1)-HRD1 arm under conditions of reticulum stress [30] (Figure 2). Finally, indirect regulation may occur through the modulation of miRNAs controlling KEAP1 and CUL3 [31].

## 3. NRF2 and Its Antioxidant Role

NRF2 deficiency causes an increase in ROS and oxidative stress in a cell type-dependent manner. Thus, mouse embryonic fibroblasts (MEFs) isolated from NRF2 knockout (KO) animals do not show increased ROS formation, in contrast to glioneuronal cells [32]. Similar discrepancies in oxidative status can be also found between organs, with the liver having the highest oxidative burden, whereas the aorta is protected from oxidative damage by increased levels of nitric oxide (NO) and mono-nitrogen oxides (NOx) [33]. The relationship between NO and oxidative stress could constitute a compensatory mechanism protecting endothelial cells without NRF2 from oxidative stress damage. The protective effect may also depend on NADPH oxidase (NOX)-4 levels, as occurs in fibroblasts from NRF2 KO mice that do not show increased ROS formation [34].

Mitochondria are responsible for more than 90% of oxygen utilization. Although most oxygen undergoes a complete reduction to water at the level of cytochrome oxidase, partial reduction accompanied by the generation of ROS can also occur, the most common being O_2^•−^_ [2]. As aforementioned, NRF2 drives the expression of the main antioxidant enzymes in the cell for oxidative stress detoxification. SOD catalyzes the conversion of O_2^•−^_ into H_2_O_2_ and molecular oxygen. Subsequently, the enzyme catalase (CAT), GPx and/or a (TRX)-dependent peroxiredoxin (PRX) reduce H_2_O_2_ to water [35]. Concerning the SOD enzyme, there are three isoforms encoded by three members of the SOD family in humans, mammals and most chordates: *SOD1* (cytoplasmic Cu-ZnSOD), *SOD2* (mitochondrial MnSOD), and *SOD3* (extracellular Cu-ZnSOD). SOD1 is responsible for regulating basal levels of superoxide-derived oxidative stress produced in both the cytosol and mitochondria [36]. SOD2 is inducible by oxidative stress, hyperoxia, environmental pollutants, and inflammatory cytokines [37], whereas SOD3 is responsible for protection against exogenous and environmental stresses, which can come from cigarette smoke, traffic exhaust emissions, solar radiation, and even food [38].

Under stress conditions, the ER plays a key role in the generation of ROS that dictate the fate of protein folding and secretion [39]. In peroxisomes, CAT is the main oxidoreductase responsible for the metabolism of H_2_O_2_ produced after the action of peroxisomal oxidases and xanthine oxidase, which generate ROS [3]. At the plasma membrane, ROS generation begins with the rapid uptake of oxygen, the activation of NOX, and the production of the O_2^•−^_ that SOD then rapidly converts to H_2_O_2_ [40]. To limit oxidative damage, the protein PKR-like endoplasmic reticulum kinase (PERK) and IRE1 phosphorylate and activate NRF2. In addition, reticulum or mitochondrial stress stimulates activating transcription factor 4 (ATF4) that cooperates with NRF2 to upregulate cytoprotective genes [41,42].

## 4. NRF2 beyond Its Antioxidant Role

In addition to its main role modeling the antioxidant response, NRF2 is involved in many other cellular pathways, at both physiological and pathological levels.

### 4.1. NRF2 in Autophagy and Protein Degradation

**Figure 3:**
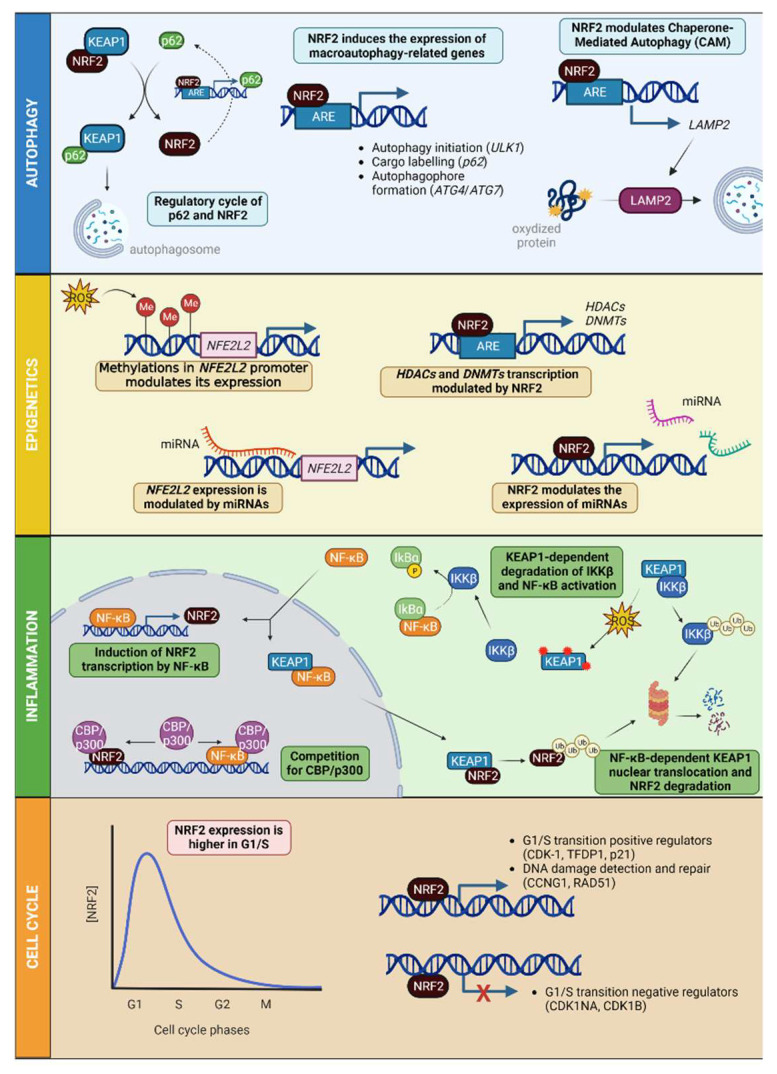
NRF2 beyond its antioxidant role: autophagy is able to potentiate NRF2 activity by tagging KEAP1 for degradation with p62. Furthermore, NRF2 is able to promote both macroautophagy and chaperone-mediated autophagy (CAM) by promoting the expression of genes related to both pathways. From an epigenetic point of view, NRF2 expression is regulated by methylation marks and miRNAs, as well as NRF2 modulates the expression of HDACs and DNMTs, in addition to miRNAs. NRF2 is highly involved with inflammatory processes; NF-κB potentiates NRF2 expression; both NFκB and NRF2 compete for CBP/p300 binding; NRF2 is degraded in an NF-κB-dependent manner; NF-κB is activated under oxidative stress in a KEAP1-dependent manner. Cell-cycle regulation is also mediated by NRF2. Its expression peaks during G1/S phases, whereas it decreases to its minimum in G2/M. The G1/S transition is regulated by the expression of promoter genes and the repression of negative regulators by NRF2, in addition to the enhancement of the expression of DNA damage detection and repairing genes by NRF2. Abbreviations: LAMP2, receptor lysosomal-associated membrane protein 2A; Me, methylation; miRNA, microRNA; NF-κB, nuclear factor kappa-light-chain-enhancer of activated B cells; CBP/p300, CREB-binding protein; IKKβ, inhibitor of nuclear factor kappa-B kinase subunit beta; IkBα, inhibitor of nuclear factor kB alpha; ROS, reactive oxygen species. Created with BioRender.com (accessed on 11 July 2023).

Autophagy is a transcriptionally controlled process that ensures the degradation of misfolded, oxidized or altered proteins to maintain cellular proteostasis. The most prevalent form of autophagy is macro-autophagy, and during this process, the cell forms a double-membrane sequestering compartment termed the phagophore, which matures into an autophagosome. Following delivery to the vacuole or lysosome, the cargo is degraded, and the resulting macromolecules are released back into the cytosol for reuse. Given the role of NRF2 as a sensor of oxidative stress, it is not surprising that a connection is established between this factor and macro-autophagy. In fact, the cargo protein sequestosome-1/ubiquitin-binding protein p62 (SQSTM1/p62) interacts with KEAP1 by directing it to the phagophore (precursor of the autophagosome), releasing NRF2 from its inhibition. In addition, *SQSTM1* contains an ARE element and thus can be transcriptionally regulated by NRF2 creating a regulatory cycle [43,44]. In addition to SQSTM1/p62, NRF2 is able to activate genes linked to autophagy initiation, cargo recognition, autophagosome formation, elongation, and autolysosome clearance [45] (Figure 3).

Proteostasis would thus be regulated in periods of nutritional stress (through Transcription factor EB, TFEB, and forkhead box proteins, FOXO3/FOXO3A) [46] or oxidative stress (through NRF2) [45].

NRF2 also participates in the control of oxidized protein degradation. These oxidized proteins can be eliminated by a type of autophagy called chaperone-mediated autophagy (CMA), characterized by the presence of the receptor lysosomal-associated membrane protein 2A (LAMP2A) (Figure 3). The control of NRF2 at the transcriptional level is due to the presence of 2 ARE elements in the *LAMP2A* promoter [47]. This regulation is independent of the effect of NRF2 on the macroautophagic activity [45].

### 4.2. NRF2, Epigenetics and miRNAs

Epigenetic modifications modulate gene expression, allowing cells to adapt to the environment through histone modifications, DNA methylation, or the modulation of specific levels of miRNAs. These epigenetic changes control transcription, cell cycle, autophagy, DNA repair, stress response, and senescence [48,49]. In this context, NRF2 acts by inducing epigenetic changes in the same way that epigenetic changes are capable of modulating their own expression. Oxidative stress can suppress *NFE2L2* expression through hypermethylation of CpG islands present in the *NFE2L2* promoter, as has been shown in prostate tumors [50]. In contrast, in colorectal cancer, *NFE2L2* repression is associated with an increased frequency of demethylation [51]. Furthermore, miRNAs capable of regulating *NFE2L2* were identified in the context of cancer [52] and cardiovascular or neurodegenerative diseases [53,54]. Recently, thanks to the analysis of embryonic fibroblasts derived from NRF2-deficient mice, or with the use of activators that modulate the KEAP1 and GSK3 pathways in hippocampal neurons, it was shown that NRF2 can in turn regulate epigenetic mechanisms by controlling the expression of *HDAC1*, *SIRT1,* and DNA (cytosine-5)-methyltransferases (*DNMTs*) that have ARE elements in their promoters [31]. The modulation of oxidative stress-associated miRNAs (redoximiRs), such as miR-27c-3p, miR-27b-3p, miR-128-3p and miR-155-5p, was also demonstrated [55]. These redoximiRs are able to modulate NRF2 levels [56]; in turn, NRF2 is able to modulate the action of these miRNAs through direct degradation [57] or by modulating their biogenesis [31] (Figure 3).

### 4.3. NRF2 Interaction with NF-κB and Inflammation

Oxidative stress-mediated signaling mechanisms are involved in inflammation and tissue injury. Elevated ROS production as a result of inflammatory signaling can mediate canonical nuclear factor kappa-light-chain-enhancer of activated B cells (NF-κB) activation and inflammatory gene induction; proteasome activity; antioxidant gene transcription; inflammasome activation; and cytokine secretion. There are at least two separate pathways for NF-κB activation. The canonical pathway is triggered by Toll-like receptors (TLRs) and pro-inflammatory cytokines such as tumor necrosis factor alpha (TNFα) and interleukin (IL)-1, leading to the expression of nuclear factor NF-κB p65 subunit (*RELA* gene), which regulates the expression of pro-inflammatory and cell survival genes [58]. The alternative NF-κB pathway is activated by lymphotoxin beta (LTβ), CD40 ligand (CD40L), B-cell activating factor (BAFF), and the receptor activator of nuclear factor kappa-B ligand (RANKL), and results in the activation of transcription factor RelB/p52 complexes (*RELB* gene) [59]. These pathways are characterized by the requirement of the inhibitor of NF-κB (IkB) kinase (IΚK) subunits. IKKβ regulates the activation of the canonical pathway through phosphorylation of IkBs and requires the IKKγ/ΝΕΜO, whereas IKKα is required for activation of the alternative pathway through phosphorylation of the IKKγ subunit and the processing of p100, the precursor of p52. These events are subjected to redox control through several modes of IkBα regulation [60], but one that has recently been described involves the regulation of IKKβ stability by KEAP1. Like NRF2, KEAP1 binds to IKKβ for ubiquitination and proteasomal degradation. In the presence of ROS, KEAP1 is inhibited and IKKβ is stabilized, phosphorylating IkBα and leading to its degradation and thus the upregulation of NF-κB [61] (Figure 3). Studies using animal models or different cell types, such as microglial cells and monocytes, suggest that upregulation of NRF2 decreases NF-κB-regulated pro-inflammatory and immune responses. Overexpression of NRF2 was also shown to inhibit Ras-related C3 botulinum toxin substrate 1 (RAC1)-dependent NF-κB activation [62]. Additionally, NF-κB may promote interaction of HDAC3 with MAF proteins, therefore preventing their dimerization with NRF2 [20].

At the transcriptional level, NF-κB activates NRF2 expression due to the existence of several functional binding sites in the promoter region of the *NFE2L2* gene, thus inducing a negative feedback loop [63]. In addition, both NF-κB and NRF2 transcription factors compete for binding of the CBP/p300 coactivator. On the other hand, NF-κB binds to KEAP1 and translocates it to the nucleus, thus favoring NRF2 ubiquitination and degradation [64] (Figure 3). Finally, E3 ligase β-TrCP labels both IkBα and NRF2 for proteasomal degradation [20], and therefore may lead to increased NF-κB activity. Thus, NRF2 and NF-κB influence each other to control antioxidant and inflammatory responses.

### 4.4. NRF2 in Cell Cycle

Recently, Lastra et al. showed that NRF2 levels oscillate during cell-cycle progression, reaching a peak in G1/S and being minimal in G2/M [65] (Figure 3). The decrease in NRF2 levels leads to an increase of cells in G1 as they are not ready to enter the S-phase with subsequent cycle arrest at the G1/S restriction point. The role of NRF2 in cell-cycle progression occurs both at the transcriptional level and through mechanisms independent of NRF2-binding to ARE elements in their target genes. Thus, NRF2 is required for the expression of positive regulators (cyclin-dependent kinase (CDK)-2, and the transcription factor Dp-1, TFDP1) and the repression of the negative regulators cyclin dependent kinase inhibitor 1A (CDKN1A), and cyclin dependent kinase inhibitor 1B (CDKN1B) of the G1-S transition. In addition, it is also involved in the optimal expression of genes involved in DNA damage detection (cyclin-G1, CCNG1) and repair (DNA repair protein RAD51 homolog 1, RAD51) [65] (Figure 3). On the other hand, as has been demonstrated in NRF2-deficient mice, a reduction in the levels of this transcription factor releases KEAP1 from its binding and allows it to bind to other proteins such as p21 at the G1/S transition [66]. Embryonic fibroblasts from NRF2-deficient mice showed decreased cell growth and a shorter half-life compared with MEFs from wild-type mice [67].

## 5. NRF2 in Liver Pathology

Liver diseases contribute to more than 3.5% of deaths in developed countries [68]. One of the main functions of the liver is the detoxification and elimination of potential harmful xenobiotics. Most of the enzymes in charge of those functions are induced by NRF2 [69]; therefore, the importance of this transcription factor in liver pathophysiology is not surprising. A summary of the role of NRF2 in different models of liver pathology can be found in Table 1.

### 5.1. NRF2 in Liver Inflammation

Activation of the inflammasome has been linked to the oxidative stress associated with various liver pathologies. ROS are necessary for the activation of the NLR family pyrin domain containing 3 (NLRP3) in all its stages, as well as for the formation of pores in the plasma membrane leading to the release of pro-inflammatory cytokines [70]. NLRP3 inflammasome is a complex of proteins that assembles in the cytoplasm in response to inflammatory stimuli and leads to the activation of the enzyme caspase-1, which is responsible for the cleavage and activation of the precursor forms of two important inflammatory cytokines, IL-1β and IL-18 [71]. In this sense, NRF2 inhibits the expression of pro-inflammatory cytokines by blocking the recruitment of RNA Pol II [72]. In addition, increased ROS oxidize thioredoxin that is no longer bound to thioredoxin-interacting protein (TXNIP) which can bind to NLRP3, activating the inflammasome pathway [73]. In the liver, NLRP3 upregulates KEAP1, increasing hepatic fibrogenesis by decreasing NRF2 activation, and in turn increasing ROS levels and pyroptosis, further exacerbating fibrosis [74].

### 5.2. NRF2 and Insulin Resistance

It was shown that the enhancement of NRF2 activity in KEAP1 KO mice increased the phosphorylation of AMPK in the liver, as well as insulin-signaling in skeletal muscle, resulting in a substantial improvement of glucose tolerance [75]. Moreover, NRF2 KO mice exhibit increased insulin sensitivity, which was attributed to ROS- mediated inhibition of PTP1B that antagonizes insulin-signaling [76]. Thus, NRF2 plays a complex role in tissue-specific insulin resistance and additional research is needed to elucidate the full array of NRF2 functions in tissues involved in the control of whole-body glucose homeostasis.

### 5.3. NRF2 and Liver Regeneration

The liver is one of the few adult mammalian organs that retains a remarkable ability to regenerate itself. Resection of up to 70% of the liver mass via partial hepatectomy leads to compensatory growth from the intact tissue and fully restores organ size. NRF2 is required for the timely M-phase entry of replicating hepatocytes by ensuring proper regulation of cyclin A2 and the Wee1/Cdc2/cyclin B1 pathway during liver regeneration [77]. Cell regeneration is diminished in hepatectomized NRF2-KO mice, which is associated with increased oxidative stress, reduced insulin/insulin growth factor-1 signaling [78], and reduced expression of the gene encoding a hepatotropic factor, an augmenter of liver regeneration [79]. The liver may also regenerate following injury by exogenous and/or endogenous agents (e.g., alcohol, hepatitis B/C viruses, and fatty acids) that cause hepatocyte death. This process is characterized by an inflammatory reaction and extracellular matrix (ECM) synthesis/remodeling. However, if the damaging insult persists, the tissue will be repaired instead of regenerated, resulting in the excessive scarring known as fibrosis. Cholesterol affects the balance between hepatocyte proliferation/regeneration and liver tissue fibrosis in the attempt to restore organ homeostasis [80]. Cholesterol induces liver regeneration and activation of NRF2 and hypoxia-inducible factor (HIF)-1α to increase hepatocyte protection against bile acids [81] and induce hepatocyte proliferation. On the contrary, bile acids promote liver injury via their detergent and cytolytic action and by inducing ER stress and mitochondrial damage [82]. They also initiate the transdifferentiation of hepatic stellate cells (HSCs) into myofibroblasts, which ultimately leads to fibrosis [83].

### 5.4. NRF2 in Acute Liver Injury

Acute liver failure (ALF) is a serious liver injury characterized by oxidative stress, inflammatory response, and apoptosis produced by numerous factors, such as viral infection, alcohol, drug abuse, and metabolic and autoimmune disorders. Several chemicals and pathogens such as thioacetamide (TAA), LPS, concanavalin A (ConA) or acetaminophen (APAP) commonly induce an acute hepatotoxicity [84]. Currently, no effective treatment options are available for ALF except for liver transplantation, so there is an urgent need to find an effective therapy for the treatment of the liver injury. NRF2 is a regulator of cellular defense pathways against the oxidative stress caused by xenobiotics; thus, its pathway might be potentially targeted as a pharmacological approach against ALF.

For example, ConA and TAA induce oxidative stress; therefore, inhibition or deletion of KEAP1 that consequently induces NRF2 activity as protective strategies ahead of liver injury are of interest [85,86,87,88,89]. In a different way, APAP induces cell death through apoptosis, necrosis and ferroptosis [90,91]. Ferroptosis, in particular, is triggered by lipid peroxidation caused by the activation of CYP4204E1 by APAP, which causes a drop in GSH levels and an accumulation of ROS [92]. In NRF2-KO mouse models, oxidative stress is exacerbated after treatment with both APAP and ConA, in agreement with the crucial role of NRF2 in detoxifying the tissue after ALF [93,94]. The administration of natural components extracted from plants in models of ALF reduced cell death and tissue damage, increasing the hepatic capacity to eliminate xenobiotics by controlling the expression of the multidrug resistance protein 3 (MRP3) transporter [95]; upregulating the AMPK/GSK3β/NRF2 signaling pathway [96]; or modifying KEAP1 cysteines, thus blocking NRF2 degradation [97].

LPS is a component of Gram-negative bacteria that can stimulate various signaling pathways such as mitogen-activated protein kinases (MAPKs) and NF-κB signaling pathways in Kupffer cells. These pathways ultimately lead to the production of proinflammatory cytokines and chemokines, which enhance local inflammation and immune-cell infiltration in the liver tissue, inducing hepatocyte pyroptosis and liver injury [98]. During sepsis caused by LPS, NRF2 sumoylation is inhibited, leading to a decrease in the hepatic GSH levels causing cellular damage [99].

### 5.5. NRF2 in MAFLD/NASH

Metabolic-associated fatty liver disease (MAFLD) is defined as a condition where hepatic fat accumulation exceeds 5% of the liver’s weight without alcohol consumption (<30 g per day). It covers a wide spectrum of pathological conditions, extending from simple steatosis (NAFLD, deposit of fat in hepatocytes) to nonalcoholic steatohepatitis (NASH, characterized by the presence of 5% hepatic steatosis and inflammation with hepatocellular damage, with or without fibrosis), cirrhosis, and ultimately leading to hepatocellular carcinoma [100]. Insulin resistance seems to play a key role in the initiation and progression of the disease from simple fatty liver to advanced forms due to an increase of hepatic lipogenesis and a reduction of free fatty acid degradation [101]. This alteration is followed by the second hit of oxidative stress [102,103], which induces an increase in pro-inflammatory cytokines and the activation of NF-κΒ, which in turn activates the hepatic stellate cells, the subsequent fibrosis, and damage to the DNA, with a failure in the synthesis of exogenous antioxidants [104].

A microarray analysis of mouse hepatic gene expression revealed that pharmacologic and genetic activation of NRF2 suppresses key enzymes involved in lipid synthesis and reduces hepatic lipid storage [105]. NRF2 appears to protect the liver against steatosis by inhibiting lipogenesis and promoting fatty acid oxidation. This may be explained by the activation of ARE-containing transcription factors that regulate adipocyte differentiation and adipogenesis and by the protection against redox-dependent inactivation of metabolic enzymes [9]. In the liver, triglyceride synthesis is regulated by the nuclear transcription factor-liver X receptor-α (LXRα) and its downstream gene *SREBF1*, encoding the transcription factor sterol regulatory element binding 1c (SREBP-1c), which induces the expression of lipogenic genes such as acetyl-coenzyme (Co) A carboxylase (*ACACA*) and fatty acid synthase (*FASN*). Some studies have reported that NRF2 activation inhibits LXRα activity and LXRα-dependent liver steatosis through the farnesoid X receptor (FXR)- small heterodimer partner (SHP) signaling pathway. Moreover, NRF2 activator inhibits SREBP-1c and lipogenic genes by promoting deacetylation of FXR and inducing small heterodimer partner, which accounts for the repression of LXRα-dependent gene transcription, protecting the liver from excessive fat accumulation [106].

Liao et al. showed that NRF2 activation through the PI3K/AKT signaling pathway significantly enhances hepatocellular antioxidant capacity and relieves mitochondrial dysfunction by inhibiting NOX2 activation in mice fed a HFD, suggesting that PI3K/AKT/NRF2 signal transduction plays a role in the regulation of hepatocellular oxidative damage [107]. Furthermore, hesperetin, (3′,5,7-trihydroxy-4′-methoxyflavanone) a major bioflavonoid in citrus fruits, can trigger NRF2-mediated antioxidative processes and suppress fatty acid-induced ROS overproduction, leading to the attenuation of NF-κB activation and thus the inhibition of hepatic inflammation in NAFLD progression. In addition, hesperetin demonstrates the interrelationship between the antioxidative and anti-inflammatory effects in protecting against NAFLD [108].

Mechanisms underlying liver fibrosis include the activation of both hepatic stellate cells and Kupffer cells, resulting in functional and biological alterations [109]. It was also demonstrated that NRF2 deficiency induces the activation of stellate cells and exaggerates the progression of carbon tetrachloride (CCl_4_)-induced hepatic fibrosis in mice [110]. NRF2 deficiency in hepatocytes dampens the cellular antioxidant response and allows for the increased expression of pro-inflammatory genes, including *IL-6*, *IL-1b* and *TNF* [111]. NRF2 attenuates liver fibrosis due to the disruption of Janus kinase (JAK) 2/Signal transducer and activator of transcription 3 (STAT3) signaling and the higher expression of suppressor of cytokine signaling 3 [110]. Furthermore, NRF2-mediated inhibition of the transforming growth factor beta (TGFβ) signaling in stellate cells may help to decrease liver fibrosis [112].

The role of hepatocyte growth factor (HGF)/mesenchymal epithelial transition factor (c-met) axis in liver pathophysiology has been extensively investigated with a particular emphasis on aspects regarding liver regeneration, hepatocyte proliferation, and apoptosis [113]. The disruption of c-met functionality aggravates the onset of NASH through the impairment of mechanisms regulating cell sensitivity to lipotoxicity, ROS production, and cell proliferation. In particular, data emerging from genomic array analysis clearly indicated an aberrant regulation of a pattern of genes responsible for increased pro-oxidant environment, amongst them the transcription factor NRF2 [114]. The generation of double mutant *c-met/Keap1^ΔHepa^* mice further demonstrated that re-establishing a functional antioxidant activity completely reversed the accelerated pathological conditions observed in single *c-met^ΔHepa^* mice. In particular, the reduction of oxidative stress was accompanied by a decrease in the above-mentioned pro-oxidant systems, CYP2e1, CYP4a10, and NOX2 expression [115]. The amelioration of the redox balance occurred concomitantly with a reduced hepatic accumulation of triglycerides related to the inhibition of the LXR-dependent lipogenic program induced by NRF2 [116].

### 5.6. NRF2 in ALD

The spectrum of alcohol liver disease (ALD), a leading cause of mortality within liver disorders, refers to hepatic steatosis, alcoholic hepatitis, fibrosis, cirrhosis, and eventually hepatocellular carcinoma in some cases [117]. Chronic alcohol consumption increases ROS production, ER stress, disruption of lipid metabolism and mitochondria dysfunction, decreases antioxidant levels, and enhances oxidative stress, especially in the liver as the main organ in which alcohol is metabolized. Alcohol dehydrogenase is the enzyme that transforms alcohol to acetaldehyde, a profibrogenic factor that induces GSH depletion; the generation of ROS and acetaldehyde adducts; and lipid peroxidation [118]. If dehydrogenase becomes saturated, alcohol continues to be oxidized through microsomal CYP2E1, generating more adducts, ROS, and free radicals [119]. Both homocysteine activation and CYP2E1 expression increase the expression of NRF2 and its target genes, especially *HMOX1* [120]. Thus, NRF2-deficient mice have increased mortality as a result of increased lipogenesis, glutathione depletion, and increased inflammation [121]. Paradoxically, NRF2 activation also contributes to the pathogenesis of ALD via the upregulation of hepatic very low-density lipoprotein receptor (VLDLR) levels [122]. Ethanol administration decreases mitochondrial glutathione concentrations in NRF2-KO mice but not in mice where NRF2 expression is enhanced [123]. Compared with the dramatic phenotype in global NRF2-KO mice, the contribution of hepatic NRF2 seems to be smaller than in the liver-specific NRF2(L)-KO mouse model. There is a clear indication that NRF2 in the central nervous system plays a major role in the sensitivity to ethanol-induced lethality in the global NRF2-KO mice [124].

### 5.7. NRF2 in Hepatocellular Carcinoma (HCC)

The role of NRF2 during HCC development is controversial. A study analyzing several HCC human samples recently reported that mutations in either KEAP1 or NRF2 occur in approximately 12% of all cases [125], implicating that an active NRF2 pathway could induce or drive HCC development. Mutations in the tumor suppressor PTEN cause a downregulation of the PTEN/GSK-3/β-TrCP pathway through increase in phosphatidylinositol-3-kinase (PI3K)-AKT signaling, preventing the proteasomal degradation of NRF2, thus being implicated in NRF2 activation [20]. It was shown that persistent NRF2 activation contributes to different pro-oncogenic pathways. First, elevated NRF2 levels may promote cancer-cell proliferation [126]. Second, cancer cells with elevated NRF2 levels are less sensitive to chemotherapeutic agents [127] and ionizing radiation [128]. However, in an inflammation-driven murine model of liver carcinogenesis (NEMO^ΔHepa^), liver-specific activation of NRF2 (NEMO^ΔHepa^/KEAP1^ΔHepa^) showed reduced apoptosis as well as a dramatic downregulation of genes involved in cell-cycle regulation and DNA replication. Consequently, double KO mice NEMO^ΔHepa^/KEAP1^ΔHepa^ displayed decreased fibrogenesis, lower tumor incidence, reduced tumor number, and decreased tumor size [74]. Therefore, the NRF2/KEAP1 pathway has the role of a double-edged sword, and NRF2 inducers act to protect normal cells from carcinogens, whereas NRF2 inhibitors act to suppress the proliferation of cancer cells that evolved from persistent NRF2 activation due to mutations.

### 5.8. NRF2 in Ischemia-Reperfusion Injury

Ischemia-reperfusion (I/R) injury (IRI) is a pathology that occurs in situations of transplant and/or resection. The tissue remains without oxygen and nutrients, causing dysfunction, injury, and cell death, varying according to the degree and time of ischemia. Revascularization and restoration of blood flow is the only therapeutic approach, but paradoxically, it exacerbates damage to the tissue. Hepatocellular death is characterized mainly by ROS-induced necrosis and innate pro-inflammatory cytokine-mediated apoptosis in the IRI liver [129]; therefore, the role of NRF2 is crucial in this pathology and of high interest as a therapeutic target.

Several experimental animal studies have highlighted the possibility of using NRF2 modulation for IRI attenuation in liver transplantation. Thus, Ahmed et al. have recently shown that NRF2 expression is higher in human liver allografts that exhibit significantly better clinical parameters than NRF2-deficient livers [130]. Ex vivo preservation by mechanical perfusion (MPN) offers a unique opportunity not only to preserve allografts, but also to consider the addition of compounds that could improve allograft viability and quality and exert a hepato-protectant effect. The modulation of NRF2 activity in organs connected to MPN decreases vascular inflammation and periportal CD3^+^ T-cell infiltration; increases cellular vacuolation; improves lactate clearance; and reduces transaminase alterations after MPN, pointing to NRF2 as a predictive biomarker [131]. The use of Institut Georges Lopez-1 (IGL-2) preservation solution based on polyethylene glycol 35kDa (PEG35) and GSH improved mitochondrial function and reduced oxidative stress during the cold preservation of livers to be transplanted. The authors demonstrated that the effect of the IGL-2 solution was due to changes in the NRF2/HO-1 pathways, reduction of the NRLP3 inflammasome pathway, and increased mitophagy [132].

In ischemia, mitochondria are the key organelles in ROS generation. Oxygen deficiency increases the reduced state of mitochondria by increasing the NADPH/NADP+ ratio and decreasing ATP generation. NADPH is the main reducing resource of the organism, and many oxidoreduction reactions, such as the reduction of oxidized glutathione (GSSG) and thioredoxin, are carried out by oxidizing NADPH to NADP+. Most cellular NADPH is generated by the pentose phosphate pathway, and small amounts of NADPH are generated by the malic enzyme. NRF2 controls NADPH levels by regulating four of the key genes in NADPH synthesis (*G6PD*); phosphogluconate dehydrogenase (*PGD*); malic enzyme 1 (*MEI*); and isocitrate dehydrogenase (*IDH*), as well as by promoting metabolite reduction by activating NQO1 and NQO2 [133].

Cell apoptosis contributes to damage during hepatic I/R via Tollip-ASK1-JNK/p38 and through regulation of the inflammatory response and associated apoptosis via MAPK. NRF2 prevents apoptosis by upregulating the expression of anti-apoptotic B-Cell CLL/Lymphoma 2 (BCL-2) proteins and decreasing BCL-2-associated X protein (BAX), cytochrome c release and caspase activation [134,135,136]. Moreover, NRF2 also modulates apoptosis through binding to ARE elements in the promoters of genes encoding for the anti-apoptotic proteins BCL-2 and BCL-XL [134,137].

Activation of the NRF2 pathway is able to protect against I/R damage through the activation of yes-associated protein 1 (YAP), which, after accumulation in the nucleus, is able to facilitate the activation of genes involved in regeneration, phase II enzymes, decreased ROS production, and the infiltration of CD68+7-Ly6G+ neutrophils [138].

**Table 1:** Summary of the NRF2 role in liver pathologies.

Liver Pathology	NRF2 Regulation	Consequence of NRF2 Regulation	Reference
Inflammation	Blocking of the recruitment of RNA Pol II	Inhibition of pro-inflammatory cytokines	[72]
Insulin resistance	Increase the phosphorylation of AMPK in the liver and the insulin signaling in skeletal muscle	Improvement of glucose tolerance	[75]
Acute liver injury	Absence of NRF2 in APAP treatment activates CYP4204E1, decreases GSH levels and accumulates ROS.	Enhancement of oxidative stress	[92]
	Absence of NRF2 enhances oxidative stress when treated with both APAP and ConA	The liver is unable to detoxify xenobiotics	[93,94]
	KEAP1-dependent release of NRF2 by natural components	Reduce cell death and tissue damage by increasing hepatic detoxification capacity	[97]
MAFLD/NASH	Inhibition of lipogenesis and increase fatty acid oxidation through the activation of ARE-containing transcription factors	Improvement of steatosis	[9]
	Suppression of key enzymes involved in lipid synthesis and reduction of hepatic lipid storage		[105]
	Inhibition of LXRα activity, LXRα-dependent liver steatosis and SREBP-1c and lipogenic genes		[106]
	Inhibition of NOX-2 activation	Enhancement of hepatocellular antioxidant capacity and improvement of mitochondrial dysfunction	[108]
	Inhibition of TGFβ signaling in stellate cells	Reduction of liver fibrosis	[112]
ALD	Upregulation of hepatic very low-density lipoprotein receptor levels	Contribution to the pathogenesis of ALD	[119]
	CYP2E1 detoxifying enzyme increase NRF2 expression	Increase *HMOX1* expression	[120]
HCC	Reduction of apoptosis and downregulation of genes involved in cell cycle regulation and DNA replication	Reduction of fibrogenesis, lower tumor incidence, reduced tumor number and size	[74]
	Increased NRF2 levels may promote cancer cell proliferation	Contribution to pro-oncogenic pathways and therefore to HCC	[126]
	Elevated NRF2 levels could promote cancer cells to be less sensitive to chemotherapy and ionizing radiation		[127,128]
I/R	Regulation of the key genes in NADPH synthesis (*G6PD*, *PGD*, *MEI*, *IDH*) and activation of NQO1 and NQO2	Control of NADPH levels, glutathione metabolism, and lipid biosynthesis	[133]
	Upregulation of anti-apoptotic BCL-2 proteins and reduction of BAX, cytochrome c release and caspase activation	Prevention of apoptosis	[134,135,136]
	Activation of yes-associated protein 1 (YAP) and genes involved in regeneration (CTGF, CYR61, and ANKRD1) or phase II enzymes (MnSOD and CAT). Reduction of ROS production, and infiltration of CD68+7-Ly6G+ neutrophils	Protection against I/R damage	[138]

## 6. The Relationship between NRF2 and Cyclooxygenase 2 (COX-2) in Liver Pathology

The COX-2 enzyme catalyzes the first step in the prostaglandin biosynthesis pathway, converting the arachidonic acid present in phospholipid membranes to an unstable intermediate, prostaglandin G2 (PGG_2_), that will be further converted into the various types of prostanoids [139]. Unlike the COX-1 isoform, which is constitutively expressed in almost all cell types in the body, COX-2 is only expressed in specific cells under certain stimuli [140]. In adult hepatocytes, COX-2 expression is reduced to situations where proliferation and de-differentiation occurs, as they adopt a fetal phenotype, a stage in which they are able to express COX-2 when exposed to pro-inflammatory stimuli [141].

Despite the low expression of COX-2 in the liver, the use of non-steroidal anti-inflammatory drugs (NSAIDs) appears to be effective in reducing inflammation, so COX-2 inhibition may be beneficial in the inflammatory process. Numerous studies with COX-2 inhibitors in various models of liver injury have found positive effects in reducing inflammation [142,143,144,145]. However, long-term inhibition of COX-2 or its complete depletion appears to have adverse effects without additional benefit [146,147]. Therefore, COX-2 inhibition does not seem to be the best way to treat inflammation, as COX-2 expression may be necessary for resolution of the pathological process.

**Figure 4:**
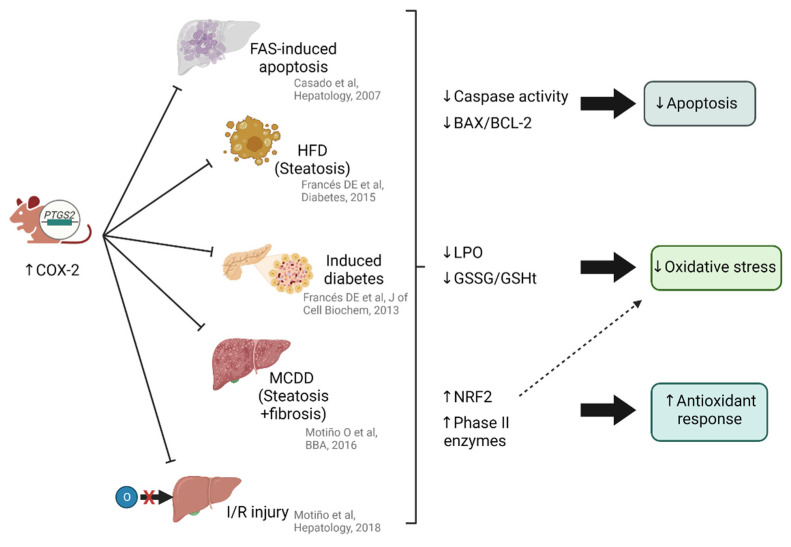
Protective effect of COX-2 overexpression in hepatocytes in different liver pathology models: the protection is due to the induction or inhibition of several pathways. Apoptotic markers, such as caspases activity and the pro-apoptotic (BAX) to anti-apoptotic (BCL-2) ratio, are decreased, leading to a decrease in apoptosis. Lipid peroxidation (LPO) and the ratio of oxidized glutathione (GSSG) to total glutathione (GSHt) decrease, which is related to a reduction in oxidative stress. Induction of NRF2 and its derived phase II enzymes enhances the antioxidant response, thus contributing to a reduction in oxidative stress. Abbreviations: HFD, high fat diet; MCDD; methionine- choline-deficient diet; I/R, ischemia-reperfusion. Data is available in the original articles [11,12,13,148,149]. Created with BioRender.com (accessed on 11 July 2023).

COX-2 has been widely demonstrated to play a role in reducing apoptosis in different liver pathologies. The pathophysiological role of COX-2 expression was analyzed in studies in which its activity was reduced by chemical inhibitors or by using knockout mouse models. However, very few studies have investigated the effects of the constitutive hepatic expression of COX-2. We generated a transgenic (Tg) mouse model carrying the human (h)COX-2 gene (*PTGS2*) under the control of the human APOE promoter and its endogenous hepatic control region (hCOX-2 Tg) (Figure 4). In an FAS-induced apoptosis model, COX-2 overexpression, specifically in hepatocytes, correlates with lower caspase activity and BAX/BCL-2 ratios compared to wild-type mice [148]. In animals with COX-2 overexpression in different stages of MAFLD, either in hyperglycemic [149], obese [11] or fibrotic [12] models, these apoptotic markers are also reduced. Along with reduced cell death by apoptosis, other damage-causing mechanisms, such as oxidative stress, are reduced. Transgenic hCOX-2 animals subjected to a methionine-choline-deficient diet show lower levels of lipid peroxidation, as well as a decreased oxidized (GSSG) to total (GSHt) glutathione (GSSG/GSHt) ratio compared to wild-type animals [12], revealing a lower generation of ROS or an enhanced antioxidant response. Very similar results are observed when COX-2 is overexpressed in animals subjected to hepatic ischemia-reperfusion [13]. In this model, the antioxidant response emerges as the main cause of reduced liver damage, driven by the master regulator of the response, NRF2. Not only is it highly expressed (gene and protein), and properly localized (in the nucleus of COX-2 expressing cells), but several phase II enzymes are highly expressed (SOD1, SOD2, HO-1). In line with the idea that the oxidative stress is the main mechanism causing damage in IRI, it was demonstrated that pre-treatment with NRF2 before ischemia-reperfusion is beneficial [150].

Hepatic human COX-2 expression protected mice from the metabolic disorder and liver injury induced by a high-fat and ethanol (HF+Eth) diet, based on the clinical significance of the coexistence of ethanol drinking and the western diet, by enhancing hepatic lipid expenditure. Hepatocyte COX-2 overexpression protected the mice from HF+Eth-induced fatty liver and metabolic dysfunction. hCOX-2 Tg mice gained less weight, showed improved glucose tolerance, serum and hepatic lipid profiles, and less fatty liver damage. The anti-lipogenic effect of hCOX-2 Tg in the HF+Eth diet animals was mediated by increasing lipid disposal through enhanced β-oxidation via elevations in the expression of the peroxisome proliferator-activated receptor (PPAR) α and γ, and increased hepatic autophagy as assessed by the ratio of the microtubule-associated proteins 1A/1B light chain 3 II and I (LC3 II/I) in hepatic tissue. Various protein acetylation pathway components, including HAT, HDAC1, SIRT1, and SNAIL1, were modulated in hCOX-2 Tg mice in either control or HF+Eth diets [151].

The interaction of NRF2 with COX-2 has already been described, for example via the end metabolite 15-deoxy-Δ12,14-prostaglandin J2 (15D-PGJ_2_), a non-enzymatic degradation product of prostaglandin D2 (PGD_2_), which induces NRF2 expression. Inhibition of KEAP1 in IRI models ensures resistance to damage through reduced inflammation, macrophage and neutrophil infiltration, apoptosis, and the promotion of antioxidant-signaling [152]. The final metabolite 15D-PGJ_2_ targets the cysteines of KEAP1 [153,154], allowing NRF2 activation, as do other anti-inflammatory molecules [155]. Moreover, 15D-PGJ_2_, in addition to NRF2 activation, induces PPARγ which will reduce NF-κB-signaling, thereby reducing inflammation [156].

The different expression patterns of COX-1 and COX-2 lead to different physiological functions. COX-1 is considered as the homeostatic stabilizer through continuous formation of prostaglandins (PGs) in the liver whereas COX-2-derived PGs are mediators of pathological conditions. Xiao et al. found that the deficiency or inhibition of enzymatic activity of COX-1 exacerbated the severity of CCl_4_-induced acute liver injury, including elevated serum aminotransferases levels, increased necrosis, and apoptosis in the liver, enhanced hepatic oxidative stress, and pro-inflammatory responses. This study of acute liver injury showed that, as observed with COX-2, PGE_2_ is the prostanoid involved in protection. However, in this case the pathway in charge of the protection is the 5-lipooxygenase pathway whose metabolites are important mediators in the inflammatory response [157].

COX-2 can also metabolize fatty acids other than arachidonic acid. For example, COX-2 converts the ω-3 fatty acids docosahexaenoic acid (DHA) and eicosapentaenoic acid (EPA) into prostaglandins, and further converts them into 13-electrophilic fatty acid oxo derivative (EFOX)-D6, and 13-EFOX-D5, respectively, via dehydrogenase and non-enzymatic reactions [158], which have anti-inflammatory and antioxidant properties. COX-2-dependent EFOXs can modify cysteines, such as those in the KEAP1 protein, and, like 15D-PGJ_2_, release NRF2 and allow its activation [159]. The molecular mechanisms involving COX-2 are not completely known, but the recent findings discussed above suggest that the intrinsic antioxidative system maintained by COX-2 and NRF2/ARE signaling constitute an important interaction in the aging process. Increasing levels of COX-2 expression during aging likely act to maintain antioxidative homeostasis as increases in ROS occur during cellular senescence, acute and chronic inflammation, and carcinogenesis [160].

**Figure 5:**
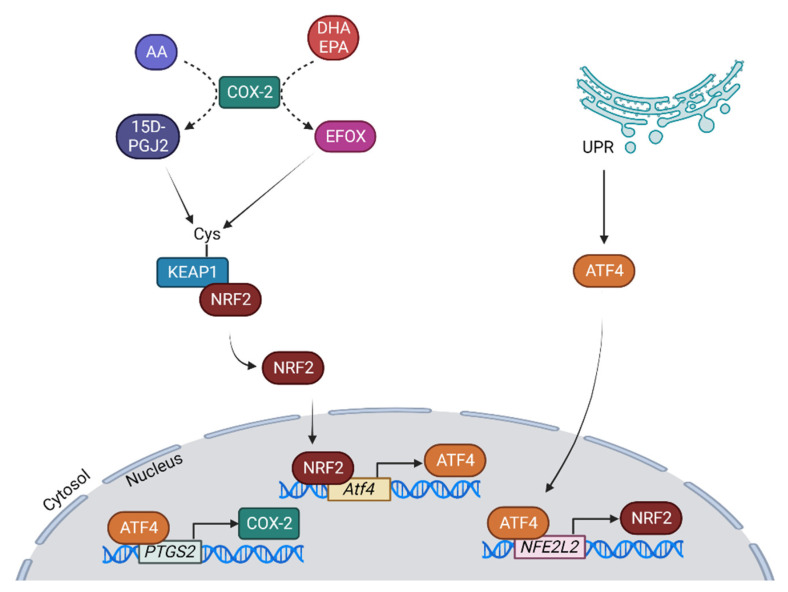
Interaction of NRF2 and COX-2 via ATF4: the end-product of the COX-2 pathway 15D-PGJ_2_ (derived from arachidonic acid (AA) metabolism) is able to modify KEAP1 cysteines, thus inducing NRF2 release. EFOX molecules, metabolized by COX-2 from DHA and EPA, are also capable of modifying KEAP1 cysteines. Once free, NRF2 translocates to the nucleus, where it induces ATF4 synthesis. ATF4 can in turn regulate NRF2 expression, in addition to inducing COX-2 expression, establishing a link between NRF2 and COX-2 expression. The ATF4 factor is also induced by the unfolded protein response (UPR), which will induce NRF2 and COX-2 expression. Abbreviations: DHA, docosahexanoic acid; EPA, eicosapentaenoic acid; EFOX, electrophilic oxo-derivative molecules. Created with Biorender.com (accessed on 11 July 2023).

Moreover, under conditions of oxidative stress, COX-2 expression is dependent on NRF2. In NRF2 KO melanoma cell lines, the *PTGS2* gene is highly downregulated, and even with H_2_O_2_ challenge, its expression is not stimulated when *NFE2L2* is silenced using siRNA [161]. NRF2 does not bind to the *PTGS2* promoter. Instead, it is the ER stress-related transcription factor ATF4 that binds to the *PTGS2* promoter and induces its expression. Jenssen and colleagues showed that in ATF4 KO, the induction of *PTGS2* expression by H_2_O_2_ is prevented, implying that ATF4 is required for COX-2 expression during oxidative stress [161]. Furthermore, in ATF4 KO, NRF2 is also reduced under H_2_O_2_ stimulation, indicating that ATF4 and NRF2 regulate each other. When NRF2 is overexpressed under control conditions, COX-2 increases, but in the ATF4 KO, COX-2 remains the same, demonstrating that NRF2-dependent COX-2 expression occurs via ATF4 [162] (Figure 5).

The relationship between ATF4, COX-2 and NRF2 has already been described. In models of tunicamycin-induced ER stress, where ATF4 appears to induce NRF2 expression [42]. In renal nephritic lupus, ER stress is enhanced, inducing ATF4 expression which will also drive COX-2 expression. This work also shows that autophagy is enhanced, a fact also observed by COX-2 overexpression in the liver IRI model [162].

Apart from the regulation of COX-2 expression by antioxidant factors, the derived prostaglandins also exert protective functions. In addition to the activation of NRF2 by 15D-PGJ_2_, the prostaglandins PGD_2_ and PGE_2_, which initially have a pro-inflammatory role, also assist in the resolution of inflammation. When they bind to their receptors on immune cells such as neutrophils, they produce a metabolic shift that results in the accumulation of cyclic AMP (cAMP) in the cytosol that causes a shift towards an anti-inflammatory profile, thus contributing to the resolution of inflammation [159].

Therefore, the canonical pro-inflammatory role attributed to COX-2 has been challenged, and this direct relationship with NRF2 poses a paradigm shift. Beyond its relationship with inflammation, it collaborates in its resolution, apparently through the antioxidant response.

## 7. Conclusions and Future Directions

Nuclear factor erythroid 2-related factor 2 (NRF2) is a regulator of several antioxidant and cytoprotective proteins and is therefore a crucial regulator of cellular responses against oxidants and oxidative stress. It has been demonstrated that in the absence of NRF2, ROS and oxidative stress damage increase inflammation and tissue injury produced by these oxidative stress-mediated signaling mechanisms. Due to this key role in oxidative stress regulation, NRF2 deficiency has been associated with several diseases, including diabetes, hyperglycemia, ischemia, atherosclerosis, acute kidney injury, and liver pathologies. In the context of the liver, NRF2 is also an important player in the induction of detoxification enzymes and transporters that aid in the elimination of harmful xenobiotics. It also has protective functions in cell metabolism, inflammation, and fibrosis. COX-2 exerts its hepatoprotective role through the modulation of antioxidant genes, control of autophagy, amelioration of inflammation, and as demonstrated recently, modulation of mitochondrial function [14]. Investigation with a COX-2 overexpression model suggests COX-2 as a potential therapeutic target to treat patients in different liver pathologies. This protection could be partially explained through the interaction with NRF2. Further research is needed to reveal the regulatory mechanisms of the KEAP-1/NRF2 pathway when COX-2 is overexpressed. Furthermore, in addition to the regulation of COX-2 expression by NRF2, the COX-2 derived prostaglandins also have a protective role. On the one hand, 15D-PGJ_2_ can activate NRF2, and, on the other hand, the prostaglandins PGD_2_ and PGE_2_ can enhance the accumulation of cAMP, a key anti-inflammatory mediator in the cytosol that leads to a shift towards an anti-inflammatory profile. Therefore, the synergistic interaction of COX-2 and NRF2 can ameliorate inflammation and contribute to its resolution through an antioxidant response in liver pathologies.

## Data Availability

Data is available in the original articles cited in the present review.

## Abbreviations

**AKT**, protein kinase B; **ALD**, alcohol liver disease; **ALF**, acute liver failure; **AMPK**, 5′ AMP-activated protein kinase; **ARE**, antioxidant response element; **BAX**, BCL-2-associated X protein; **BCL-2**, B-Cell CLL/Lymphoma 2; **β-TrCP**, F-box/WD repeat-containing protein 1A; **CAT**, catalase; **COX-2**, cyclooxygenase 2; **CUL3**, Cullin 3 **ER,** endoplasmic reticulum; **GSH**, reduced glutathione; **GSK-3**, glycogen synthase kinase 3; **HCC**, hepatocellular carcinoma; **HDAC**, histone deacetylases; **HO-1**, heme-oxygenase-1; **IKK**, inhibitor of NF-κB (IκB) kinase; **IL**, interleukin; **IRI**, ischemia-reperfusion injury; **KEAP1**, Kelch-like ECH associating protein 1; **KO**; knockout; **MAF**, small musculoaponeurotic fibrosarcoma protein; **MAFLD**, metabolic-associated fatty liver disease; **NADPH**, nicotinamide adenine dinucleotide phosphate; **NAFLD**, non-alcoholic fatty liver disease; **NASH**, non-alcoholic steatohepatitis; ***NFE2L2***, NRF2 encoding gene; **NF-κB**, nuclear factor kappa-light-chain-enhancer of activated B cells; **NLRP3**, NLR family pyrin domain containing 3; **NOX**, NADPH oxidase; **NQO1**, NAD(P)H:quinone oxidoreductase 1; **NRF2**, nuclear factor erythroid 2-related factor 2; **PG**, prostaglandin; **15D-PGJ_2_**, 15-deoxy-Δ12,14-prostaglandin J2; **PTP1B**, protein tyrosine phosphatase 1B; **RBX1**, RING-box protein 1; **ROS**, reactive oxygen species; **SIRT**, sirtuins; **SOD**, superoxide dismutase; **TGFβ**, transforming growth factor beta; **TNFα**, tumor necrosis alpha.
